# Prognostic implications of Aquaporin 9 expression in clear cell renal cell carcinoma

**DOI:** 10.1186/s12967-019-2113-y

**Published:** 2019-11-08

**Authors:** Wen-Hao Xu, Shen-Nan Shi, Yue Xu, Jun Wang, Hong-Kai Wang, Da-Long Cao, Guo-Hai Shi, Yuan-Yuan Qu, Hai-Liang Zhang, Ding-Wei Ye

**Affiliations:** 1 Department of Urology, Fudan University Shanghai Cancer Center, Shanghai, 200032 People’s Republic of China; 2 Cancer Institute, Fudan University Shanghai Cancer Center, No. 270 Dong’an Road, Shanghai, 200032 People’s Republic of China; 3 Department of Oncology, Shanghai Medical College, Fudan University, Shanghai, 200032 People’s Republic of China; 4 Department of Ophthalmology, The First Affiliated Hospital of Soochow University, Suzhou, 215000 People’s Republic of China

**Keywords:** Clear cell renal cell carcinoma, AQP9, Biomarker, Bioinformatics, Prognosis

## Abstract

**Background:**

Growing evidence has demonstrated immune reactivity as a confirmed important carcinogenesis and therapy efficacy for clear cell renal cell carcinoma (ccRCC). Aquaporin 9 (AQP9) is involved in many immune-related signals; however, its role in ccRCC remains to be elucidated. This study investigated *AQP9* expression in tumor tissues and defined the prognostic value in ccRCC patients.

**Methods:**

A total of 913 ccRCC patients with available RNA-sequence data from the Cancer Genome Atlas (TCGA) database and Fudan University Shanghai Cancer Center (FUSCC) were consecutively recruited in analyses. Differential transcriptional and proteome expression profiles were obtained and validated using multiple datasets. A partial likelihood test from Cox regression analysis was developed to address the influence of independent factors on progression-free survival (PFS) and overall survival (OS). The Kaplan–Meier method and log-rank test were performed to assess survival. Receiver operating characteristic (ROC) curves were used to describe binary classifier value of *AQP9* using area under the curve (AUC) score. Functional enrichment analyses and immune infiltration analysis were used to describe significantly involved hallmark pathways of hub genes.

**Results:**

Significantly elevated transcriptional and proteomic *AQP9* expressions were found in ccRCC samples. Increased *AQP9* mRNA expression was significantly associated with advanced clinicopathological parameters and correlated with shorter PFS and OS in TCGA and FUSCC cohorts (*p *< 0.001). ROC curves suggested the significant diagnostic and prognostic ability of *AQP9* (PFS, AUC = 0.823; OS, AUC = 0.828). Functional annotations indicated that *AQP9* is involved in the most significant hallmarks including complement, coagulation, IL6/JAK–STAT3, inflammatory response and TNF-alpha signaling pathways.

**Conclusion:**

Our study revealed that elevated *AQP9* expression was significantly correlated with aggressive progression, poor survival and immune infiltrations in ccRCC patients, and we validated its prognostic value in a real-world cohort. These data suggest that *AQP9* may act as an oncogene and a promising prognostic marker in ccRCC.

## Background

Renal cell carcinoma (RCC) is one of the most common malignant urinary tumors in the world, with an incidence rate that is increasing 2% each year, especially in developed regions [1]. The incidence and mortality rates of RCC in China are also increasing, with an estimated 66,800 new cases and 23,400 deaths in 2015 [2]. Clear cell RCC (ccRCC) is the most common and aggressive type of RCC in adults. According to the World Health Organization, ccRCC is one of the deadliest urinary tumors, with a global annual mortality rate of approximately 90,000 [3]. Although extensive research has explored the mechanism of recurrence and metastasis, the etiology and tumorigenesis of ccRCC remain unclear. A variety of indicators, such as genetic aberrations and tumor environment, have been reported to be associated with the development and progression of ccRCC [4–6]. Considering the high morbidity and mortality of RCC, it is essential to explore its causes and potential molecular mechanisms to identify potential molecular biomarkers for early diagnosis, prevention, and personalized treatment.

Aquaporins (AQPs), also called water channels, were first discovered in 1992 by Agre et al. [7], and 13 AQP family members have been identified in humans, including AQP0–12 [8]. Accumulating studies have shown that AQPs not only regulate rapid water movement in various epithelial and non-epithelial tissues [9, 10], but also participate in the pathological process of several diseases such as glaucoma, cancer, inflammation, immunity, and obesity [11]. Several AQPs are over-expressed in tumors samples and serve notable roles in cancer progression [12]. A previous study showed that *AQP1* was a unique non-invasive biomarker for screening and diagnosing malignant clear cells or papillary RCC [13]. In addition, Chen et al. also found that AQP3 promoted prostate cancer cell invasion through extracellular signal-regulated kinase 1/2-mediated MMP-3 secretion [14]. Interestingly, *AQP9* was significantly correlated with immune activity. For example, IL-7 induces glycerol channel *AQP9* expression in CD8^+^ T cells and *AQP9* is required for memory CD8^+^ T cell survival and self-renewal [15]. In addition, *AQP9* was demonstrated to promote astrocytoma cell invasion and motility via the AKT pathway [16]. Therefore, understanding of the regulation and molecular function of *AQP9* may identify potential targets for the diagnosis and treatment of ccRCC.

To investigate the differential *AQP9* transcriptional and proteomics expression and clarify the potential prognostic value in ccRCC patients, we analyzed gene expression profiles, as well as the underlying biological interaction networks and the prognostic value. We hypothesized that the possible oncogenic activity of *AQP9* may impact prognosis of ccRCC patients. Our findings may reveal potential therapeutic targets and provide insights into the molecular mechanisms of ccRCC.

## Materials and methods

### Ethics statement

All of the study designs and test procedures were performed in accordance with the Helsinki Declaration II. Study protocols were obtained by Fudan University Shanghai Cancer Center (FUSCC) (Shanghai, China) included in this work.

### Patients and transcriptional expression profile

A total of 533 ccRCC patients with available RNA-sequence data from the Cancer Genome Atlas (TCGA) database were consecutively recruited in analyses [17]. The gene expression profile was measured experimentally using the Illumina HiSeq 2000 RNA Sequencing platform by the University of North Carolina TCGA genome characterization center. Level 3 data was downloaded from TCGA data coordination center. X-tile software was utilized to take the cut-off value of mRNA expression of *AQP9*, in concordance of which overall participants were divided to two groups, respectively. Student’s t tests were used to compare differential transcriptional expressions levels of *AQP9* between paired AJCC stages or ISUP grades, marked in asterisk. The overall statistical expression difference of AJCC stages or ISUP grades was measured using One-way ANOVA test.

We next enrolled a total of 380 ccRCC patients from the Department of Urology, Fudan University Shanghai Cancer Center (FUSCC; Shanghai, China) from Aug 2009 to May 2018 in analyses. Tissue samples, including ccRCC and normal tissues, were collected during surgery and available from FUSCC tissue bank.

### Oncomine database

In this study, transcriptional expression profiles of *AQP9* in ccRCC patients were obtained from Oncomine database using Oncomine online database (http://www.oncomine.com) [18]. Difference of transcriptional expression was compared by Students’t-test. Cut-off of *p* value and fold change were as following: *p*-value = 0.01, fold change = 1.5, gene rank = 10%, Data type: mRNA.

### The Human Protein Atlas

The Human Pathology Atlas project (https://www.proteinatlas.org) contains immunohistochemistry (IHC) data using a tissue microarray-based analysis on 44 different normal tissue types, and proteome analysis of 17 major cancer types [19]. Staining intensity, quantity, location and patients’ information in patients with the respective cancer types were available online. In this study, representative proteins expressions of IHC images of *AQP9* were detected in ccRCC and normal tissues in Human Protein Atlas.

### Real-Time Quantitative PCR (RT-qPCR) analysis

Total RNA sequence was extracted using TRIzol^®^ reagent (Invitrogen Life Technologies, USA) from 380 paired tumor and para-carcinoma normal samples. Primers were diluted in ddH_2_O with SYBR Green PCR Master Mix (Applied Biosystems, Japan). Transcriptional expression was determined as the fold change of *AQP9* relative to β-Actin. PCR primers sequence for AQP9 are as follows: forward are 5′-TTGCCCAAGCTATTCTCAGTCGA-3′ and reverse are 5′-CAGAGACACCGCCAGCCACAT-3′. The *AQP9* mRNA expression was represented as ΔCt = Ct_(*AQP9*_) − ΔCt_(β-actin)_. Relative expression in ccRCC was represented using the ratio of *AQP9* expression in Tumor/Normal tissues (T/N). “Low *AQP9* expression” and “High *AQP9* expression” denote the T/N ratio of *AQP9* mRNA expression with median cutoff in FUSCC cohort.

### Immunohistochemical (IHC) staining and evaluation

Immunostaining of AQP9 was performed using a mouse monoclonal anti- AQP9 antibody (1:100, Cat. ab84828, Abcam, USA). Positive or negative staining of a certain protein in one FFPE slide was independently assessed by two experienced pathologists, and determined as follows. The overall IHC score grading from 0 to 12 was evaluated according to the multiply of the staining intensity and extent score, as previously described [20].

### Statistical analysis

Phenotype and expression profiles of hub genes in 533 ccRCC patients from TCGA were analyzed and displayed. Survival comparison between distinct mRNA expression groups of *AQP9* was analyzed in ccRCC patients. The primary end point for patients was progression-free survival (PFS), and overall survival (OS) was the secondary end point, which was evaluated from the date of first therapy to the date of death or last follow-up. The follow-up duration was estimated using the Kaplan–Meier method with 95% confidence intervals (95% CI) and log-rank test in separate curves. Univariate and multivariate analysis were performed with Cox logistic regression models to find independent variables, including age at diagnosis, age (ref. < 60 years), gender (ref. Male), pT stage (ref. T1–T2), pN stage (ref. N0), pM stage (ref. M0), AJCC stage (ref. I–II), ISUP grade (ref. 1–2) and *AQP9* expression (ref. Low). X-tile software was utilized to take the cut-off value [21]. All hypothetical tests were two-sided and *p*-values less than 0.05 were considered significant in all tests. Integrated score was identified as sum of the weight of *AQP9* and significant clinicopathological prognostic indicators.

### Protein–protein interaction (PPI) network construction

Search Tool for the Retrieval of Interacting Genes (STRING; http://string-db.org) (version 10.0) online database was used to predict PPI network of co-regulated hub genes and analyzing the functional interactions between proteins [22]. An interaction with a combined score > 0.4 was considered statistically significant.

### Functional annotations

Subsequently, the gene ontology (GO): BP (biological process), GO: CC (cellular component), GO: MF (molecular function) and KEGG pathways analyses for hub genes in this module were performed using Database for Annotation, Visualization and Integrated Discovery (DAVID; http://david.ncifcrf.gov; version 6.8) online database [23], and then visualized in bubble chart. p-value < 0.05 was considered statistically significant. Cytoscape (version 3.5), an open source bioinformatics software platform, was used to visualize molecular interaction networks [24]. ClueGO is a Cytoscape plug-in that visualizes the non-redundant biological terms for large clusters of genes in a functionally grouped network [25]. The biological process from GO and KEGG pathway analysis of hub genes was performed and visualized using ClueGO (version 2.5.3) and CluePedia (version 1.5.3), a Cytoscape plug-in that visualizes the non-redundant biological terms for large clusters of genes in a functionally grouped network [26]. Gene set enrichment analysis (GSEA) was used to predict potential hallmarks using transcriptional sequences in TCGA database. A permutation test with 1000 times was used to identify the significantly changed pathways [27]. Adj. p less than 0.01 and FDR less than 0.25 were identified as significant related genes. Statistical analysis and graphical plotting were conducted using R software (version 3.3.2).

### Immune infiltration analysis

Tumor Immune Estimation Resource (TIMER, https://cistrome.shinyapps.io/timer/) was used to perform comprehensive correlation analysis between tumor-infiltrating immune cells signatures and selected hub genes. An integrated repository portal for tumor-immune system interactions (TISIDB, http://cis.hku.hk/TISIDB/index.php) [28] was utilized to examine tumor and immune system interactions in 28 types of TILs across human cancers. The relative abundance of TILs were inferred by using gene set variation analysis based on AQP9 expression profile. Spearman’s test was used to measure correlations between AQP9 and TILs. All hypothetical tests were two-sided and p-values less than 0.05 were considered significant in all tests. All of these statistical analyses were performed in R or corresponding R packages survival and survminer.

## Results

This study consisted of four stages. We first screened and compared the mRNA expression of AQP family members in ccRCC and adjacent normal tissues in the TCGA database. We then examined the prognostic value of the expressions of the AQP family members in ccRCC and found that patients with high *AQP9* expression had poor survival. In the second stage, we assessed differential *AQP9* expression at the transcriptional and protein level according to datasets hosted on the Oncomine, TCGA and FUSCC platforms. In the third stage, survival analysis based on distinct comparison expression of *AQP9* was evaluated in the TCGA and FUSCC cohorts. In the fourth stage, significantly involved hub genes of *AQP9* were screened and corresponding functional annotations were performed.

### Clinicopathological characteristics and AQP9 expression in ccRCC patients from TCGA and FUSCC

To first assess the association of AQP family member expressions with prognosis, we obtained follow-up and transcriptional expression data from TCGA and evaluated the impact of AQP0–11 expressions on the prognosis of ccRCC patients (Additional file 1: Figure S1). It suggested that high expression of *AQP9* significantly predicted poor OS (*p *< 0.001) and PFS (*p *< 0.001).

We next examined the correlations of *AQP9* expression and clinicopathological characteristics in TCGA and FUSCC cohorts. As shown in Table 1, increased *AQP9* mRNA expression in ccRCC patients significantly correlated with advanced pT (*p *< 0.001), pN (*p *< 0.001), and pM stage (*p *< 0.001), AJCC stage (*p *< 0.001) and ISUP grade (*p *= 0.004) in the FUSCC cohort. In the TCGA cohort, increased *AQP9* mRNA expression significantly correlated with advanced pT (*p *< 0.001), pN (*p *= 0.004), and pM stage (*p *< 0.001), AJCC stage (*p *< 0.001) and ISUP grade (*p *< 0.001) (Additional file 3: Table S1).

**Table 1:** Clinicopathological characteristics baseline in relation to AQP9 expression level in FUSCC cohort

Characteristics	FUSCC cohort (N = 380)	AQP9 expression		χ^2^	*p*
		High (N = 190)	Low (N = 190)		
N (%)					
Age				0.296	0.587
< 60 years	253 (66.6)	124 (65.3)	129 (67.9)		
≥ 60 years	127 (33.4)	66 (34.7)	61 (32.1)		
Gender				0.435	0.510
Male	258 (67.9)	132 (69.5)	126 (66.3)		
Female	122 (32.1)	58 (30.5)	64 (33.7)		
BMI				0.541	0.462
< 25 kg/m^2^	231 (60.8)	112 (58.9)	119 (62.6)		
≥ 25 kg/m^2^	149 (39.2)	78 (41.1)	71 (37.4)		
pT stage				34.336	*< 0.001*
T1–T2	307 (80.8)	131 (68.9)	176 (92.6)		
T3–T4	73 (19.2)	59 (31.1)	14 (7.4)		
pN stage				14.246	*< 0.001*
N0	334 (87.9)	155 (81.6)	179 (94.2)		
N1	46 (12.1)	35 (18.4)	11 (5.8)		
pM stage				40.347	*< 0.001*
M0	310 (81.6)	131 (68.9)	179 (94.2)		
M1	70 (18.4)	59 (31.1)	11 (5.8)		
AJCC stage^a^				34.072	*< 0.001*
I–II	292 (76.8)	122 (64.2)	170 (89.5)		
III–IV	88 (23.2)	68 (35.8)	20 (10.5)		
ISUP grade				4.218	*0.040*
G1–G2	182 (47.9)	81 (42.6)	101 (53.2)		
G3–G4	198 (52.1)	109 (57.4)	89 (46.8)		

*FUSCC* Fudan University Shanghai Cancer Center, *BMI* body mass index

^a^The AJCC staging system is a classification system developed by the American Joint Committee on Cancer for describing the extent of disease progression in cancer patients. It utilizes in part the TNM scoring system: Tumor size, Lymph Nodes affected, Metastases

### Differential expression of AQP9 in ccRCC patients in multiple cohorts

**Fig. 1:**
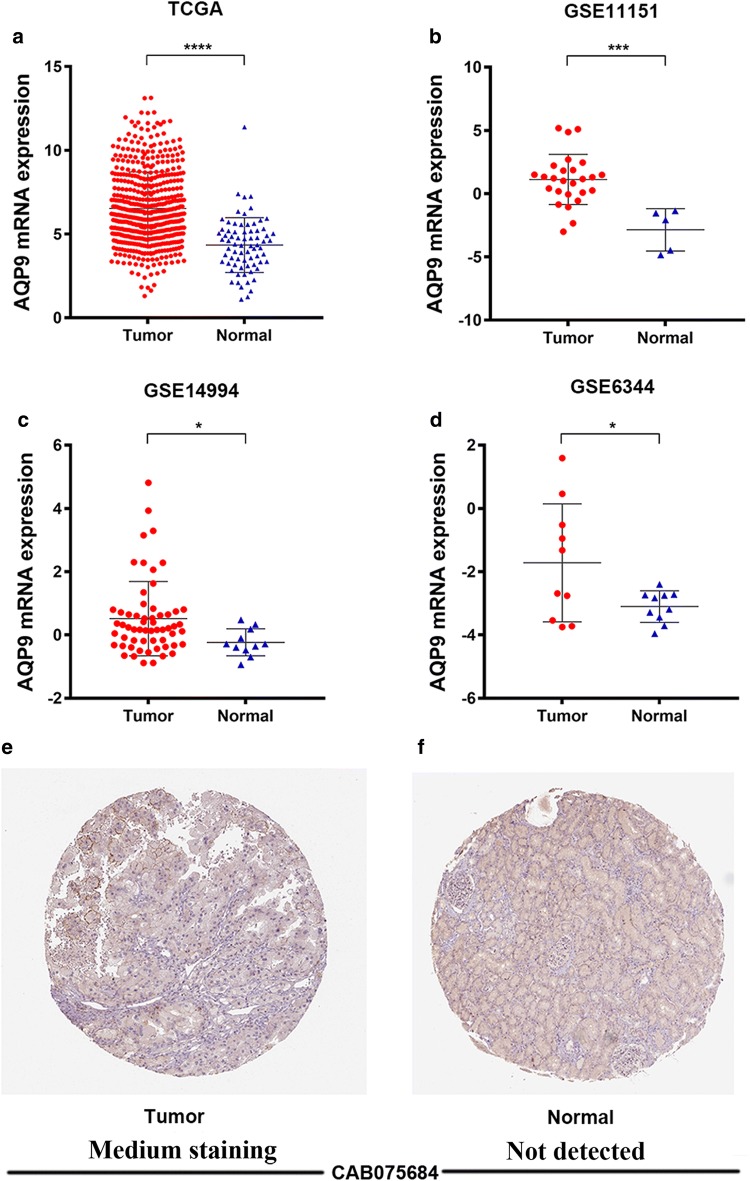
Differential *AQP9* expression in ccRCC tumor tissues and adjacent normal tissues based on multiple cohorts. **a** Transcriptional level of *AQP9* expression was found highly expressed in 533 ccRCC tissues compared with 72 normal tissues in TCGA cohort (****p < 0.0001). **b**–**d***AQP9* expression was significantly higher in ccRCC primary tumors in comparison with adjacent normal tissues in GSE11151 (Yusenko Renal dataset; ***p < 0.001) [26], GSE14994 (Beroukhim Renal dataset; *p < 0.05) [27] and GSE6344 (Gumz Renal dataset; *p < 0.05) [28] (**b**–**d**). **f** AQP9 expression is detected in ccRCC tissues while not detected in normal tissues using online database

We compared the mRNA expression of *AQP9* between ccRCC samples and adjacent normal tissues based on RNA-sequence data from TCGA and independent cohorts in silico. *AQP9* mRNA was highly expressed in 533 ccRCC tissues compared with 72 healthy tissues (*p *< 0.0001), as shown in Fig. 1a. *AQP9* expression was also significantly higher in ccRCC primary tumors in comparison with adjacent normal tissues in GSE11151 (Yusenko Renal dataset; ***p < 0.001) [29], GSE14994 (Beroukhim Renal dataset; *p < 0.05) [30] and GSE6344 (Gumz Renal dataset; *p < 0.05) [31] (Fig. 1b–d).

IHC staining indicated that AQP9 staining was not detected in normal kidney tissues, while medium levels of expression (as defined in Methods) were observed in ccRCC tumor tissues (Fig. 1e, f). Taken together, these results suggested that *AQP9* was highly expressed at transcriptional and proteomic levels in ccRCC tissues compared with normal tissues.

### AQP9 mRNA expression correlated with advanced clinicopathological parameters for ccRCC patients in TCGA cohort

**Fig. 2:**
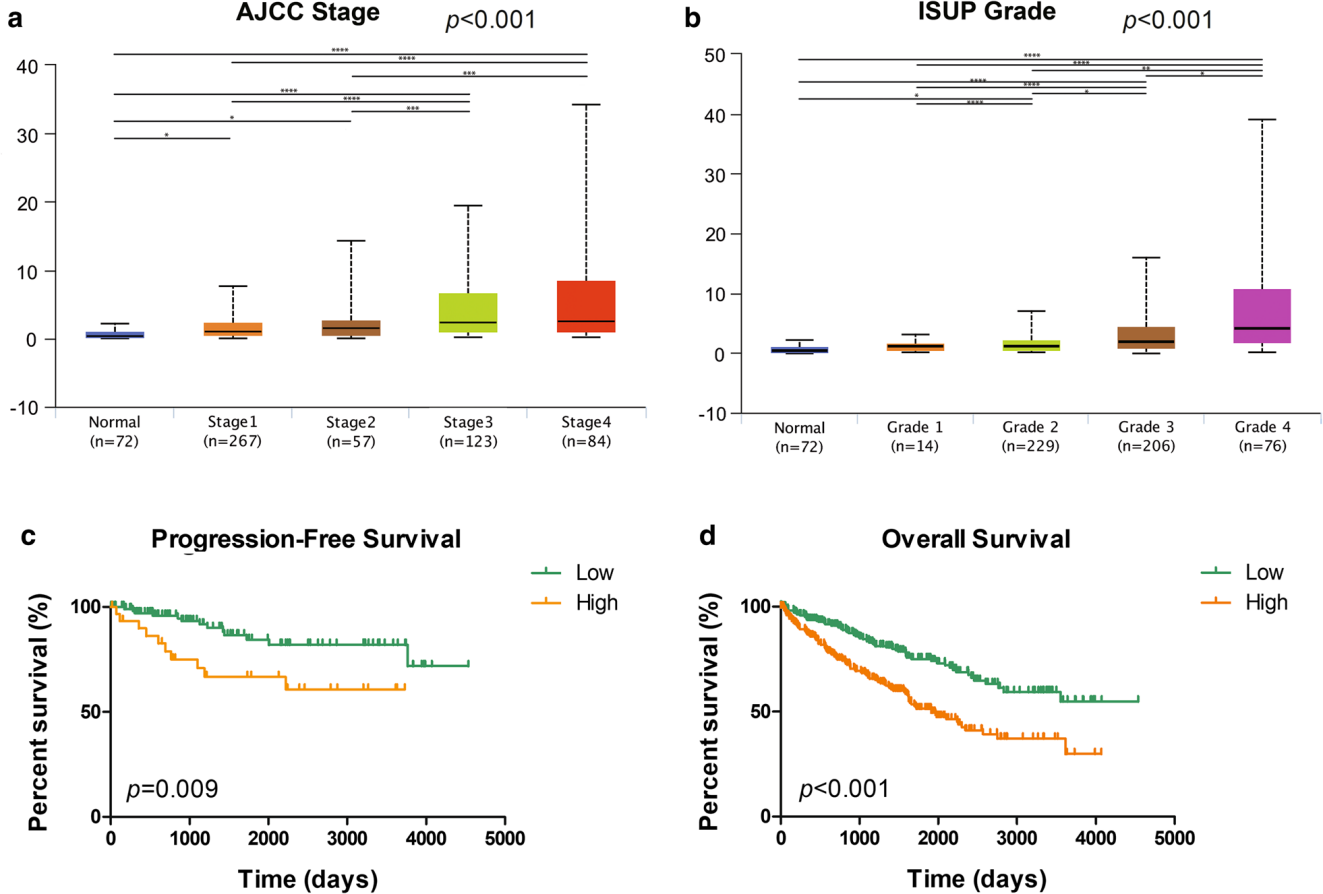
Transcriptional expressions of *AQP9* significantly correlated with advanced clinicopathological parameters and poor survival outcomes in ccRCC patients from TCGA cohort. **a** Transcriptional expression of *AQP9* was significantly correlated with AJCC stages, patients who were in more advanced stages tended to express higher mRNA expression of *AQP9*. **b** Transcriptional expression of *AQP9* was significantly correlated with ISUP grade, patients who were in more advanced grade score tended to express elevated mRNA expression of *AQP9*. Highest mRNA expressions of *AQP9* were found in stage 4 or grade 4. *p < 0.05, **p < 0.01, ***p < 0.001. **c** Survival analysis in Kaplan–Meier method indicated that *AQP9* was significantly correlated with shorter PFS (*p *= 0.009). **d** Survival curves suggested that patients with elevated *AQP9* mRNA levels showed poorer OS in 533 included ccRCC patients (*p *< 0.001)

After integrating clinicopathological and survival data from TCGA, we found significantly elevated *AQP9* mRNA expression in ccRCC samples compared with normal samples. As shown in Fig. 2a, *AQP9* mRNA expression in ccRCC samples was significantly correlated with advanced clinical stage (*p *< 0.001), and the highest *AQP9* mRNA expression was found in stage 4 cases. Figure 2b shows the relationship between *AQP9* mRNA expression and different pathological grades, and the results suggested that *AQP9* mRNA expressions were significantly correlated with pathological grade (*p *< 0.001). Similarly, the highest *AQP9* mRNA expressions were found in grade 4 cases. Survival analysis using the Kaplan–Meier method showed that elevated *AQP9* expression was significantly correlated with shorter PFS (*p *= 0.009) and OS (*p *< 0.001) in TCGA cohorts (Fig. 2c, d). Overall, elevated *AQP9* mRNA expression was significantly associated with advanced clinicopathological parameters and poor prognosis in ccRCC patients from TCGA cohort.

### Validation of elevated AQP9 expression in ccRCC tissues from the FUSCC cohort

**Fig. 3:**
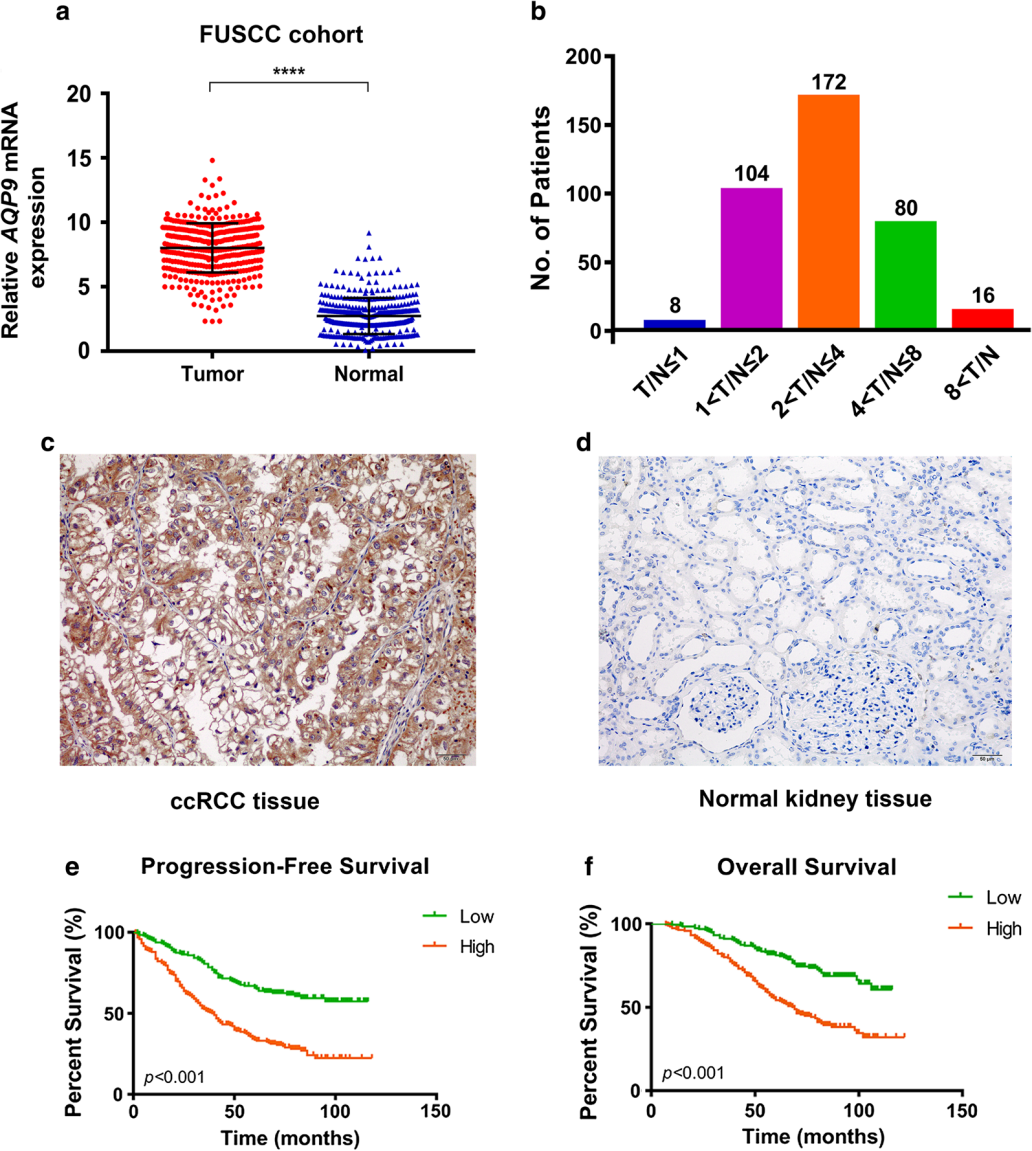
*AQP9* mRNA expression and prognostic implication in FUSCC cohort. **a** The differential *AQP9* mRNA expression in patients 380 with different T/N, which was define as the ratio of *AQP9* expression in 380 paired tumor and normal tissues. **b** Survival analysis in Kaplan–Meier method indicated that AQP9 was significantly correlated with shorter PFS (p = 0.009). **c**, **d** IHC staining indicated significantly elevated AQP9 expression in terms of density and intensity in ccRCC tissues compared with adjacent normal kidney tissues in FUSCC cohort. **e**, **f** Survival curves suggested that patients with elevated AQP9 mRNA levels correlated with poorer PFS and OS in 380 included ccRCC patients (*p *< 0.001)

To validate *AQP9* mRNA expression in ccRCC tissues, we performed RT-qPCR in 380 paired tumor and normal samples with available clinical follow-up data from FUSCC cohort. We found dramatically increased *AQP9* mRNA expression in ccRCC samples: 97.9% of ccRCC patients had higher levels of *AQP9* expression in tumor tissues than normal tissues (Fig. 3a, b). To assess the level of *AQP9* protein expression in FUSCC tumor samples, we performed IHC staining and found significant elevated AQP9 expression in terms of density and intensity in ccRCC tissues compared with adjacent normal kidney tissues in FUSCC cohort (*p *< 0.001, Fig. 3c, d).

### Cox regression analyses of TCGA and FUSCC cohorts

In univariate Cox regression analysis models, traditional prognostic factors such as pTNM stage, AJCC stage, and ISUP grade were significantly relevant to PFS (*p *< 0.05; Additional file 3: Table S2) and OS (*p *< 0.001,; Additional file 3: Table S3) in ccRCC patients in both the TCGA and FUSCC cohorts. Importantly, *AQP9* amplification markedly correlated with poor PFS (TCGA: hazard ratio [HR] = 8.141, *p *< 0.001; FUSCC: HR = 2.593, *p *< 0.001) and poor OS (TCGA: HR = 2.262, *p *< 0.001, FUSCC: HR = 2.774, *p *< 0.001).

In multivariate Cox regression analysis, traditional prognostic factors, specifically pM stage, were still relevant to PFS (TCGA: HR = 2.690, *p *= 0.043; FUSCC: HR = 2.593, *p *= 0.018; Table 2) and OS (TCGA: HR = 1.763, *p *< 0.001; FUSCC: HR = 1.895, *p *= 0.014; Table 3) in ccRCC patients. In addition, pT stage, pN stage, AJCC stage and ISUP grade were significant both in PFS (pT stage: *p *= 0.023, pN stage: *p *= 0.003, AJCC stage: *p *= 0.006, ISUP grade: *p *< 0.001) and OS (pT stage: *p *= 0.045, pN stage: *p *= 0.008, AJCC stage: *p *< 0.001, ISUP grade: *p *= 0.004) in the FUSCC cohort. Importantly, elevated *AQP9* expression was significantly associated with poor PFS (TCGA: HR = 3.443, *p *= 0.040; FUSCC: HR = 1.714, *p *= 0.001) and poor OS (TCGA: HR = 1.714, *p *= 0.026; FUSCC: HR = 1.514, *p *= 0.026) in both cohorts of ccRCC patients.

**Table 2:** Multivariate Cox logistic regression analysis of PFS in TCGA and FUSCC cohort

Covariates	TCGA			FUSCC		
	HR	95% CI	*p* value	HR	95% CI	*p* value
Age	–	–	–	1.008	0.996–1.020	0.207
Gender (ref. male)	0.563	0.157–2.022	0.379	–	–	–
pT stage (ref. T1–T2)	0.467	0.093–2.340	0.354	1.782	1.084–2.930	*0.023*
pN stage (ref. N0)	–	–	–	1.937	1.250–3.001	*0.003*
pM stage (ref. M0)	2.690	1.034–7.000	*0.043*	1.763	1.104–2.813	*0.018*
AJCC stage (ref. I–II)	4.283	0.703–26.095	0.115	2.425	1.292–4.552	*0.006*
ISUP grade (ref. 1–2)	2.269	0.817–6.301	0.116	1.812	1.330–2.471	*< 0.001*
AQP9 expression (ref. negative)	3.443	1.058–11.205	*0.040*	1.714	1.258–2.335	*0.001*

*p* value less than 0.05 are in italics

*PFS* progression-free survival, *TCGA* The Cancer Genome Atlas, *FUSCC* Fudan University Shanghai Cancer Center

**Table 3:** Multivariate Cox logistic regression analysis of OS in TCGA and FUSCC cohort

Covariates	TCGA			FUSCC		
	HR	95% CI	*p* value	HR	95% CI	*p* value
Age	1.297	0.849–1.982	0.229	1.011	0.998–1.025	0.094
pT stage (ref. T1–T2)	1.692	0.740–3.871	0.213	1.692	1.011–2.830	*0.045*
pN stage (ref. N0)	1.488	0.740–2.993	0.265	1.837	1.173–2.877	*0.008*
pM stage (ref. M0)	2.629	1.578–4.381	*< 0.001*	1.895	1.137–3.159	*0.014*
AJCC stage (ref. I–II)	1.279	0.510–3.207	0.600	3.553	1.814–6.956	*< 0.001*
ISUP grade (ref. 1–2)	1.509	0.916–2.486	0.106	1.751	1.196–2.562	*0.004*
AQP9 expression (ref. negative)	1.707	1.067–2.731	*0.026*	1.514	1.050–2.183	*0.026*

*p* value less than 0.05 are in italics

*OS* overall survival, *TCGA*: The Cancer Genome Atlas, *FUSCC* Fudan University Shanghai Cancer Center

### Prognostic value of AQP9 in TCGA and FUSCC cohorts

In TCGA cohorts, survival analysis showed that elevated *AQP9* expression was significantly correlated with shorter PFS (*p *= 0.009) and OS (*p *< 0.001). In FUSCC cohort, survival curves suggested that elevated *AQP9* mRNA levels in patients significantly correlated with poorer PFS and OS (*p *< 0.001; Fig. 3e, f). For high *AQP9* expression patients, the median PFS was 39.5 months and the median OS was 59.5 months. For low *AQP9* expression patients, the median PFS was 66 months and the median OS was 72 months. ROC curves were generated to identify the ability of the gene model to predict prognosis events.

**Fig. 4:**
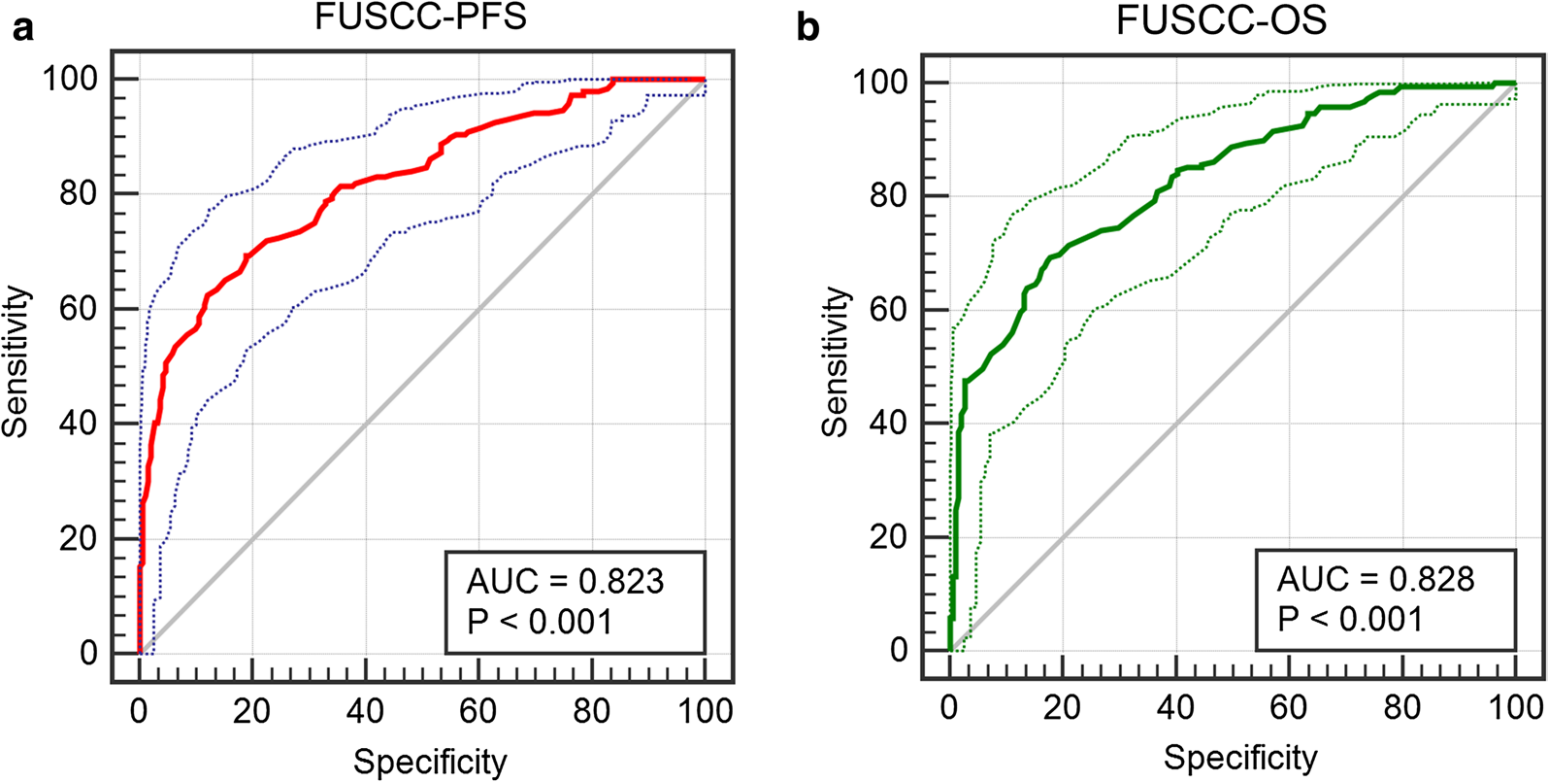
ROC curves were generated to validate the ability of the logistic model to predict prognosis. **a** The AUC index for the FUSCC-PFS were 0.823 (p < 0.001). **b** The AUC index for the FUSCC-OS were 0.828 (p < 0.001)

After integrating all the significant clinicopathological parameters and gene expression profiles in the Cox regression models (Table 2), we generated the formula: 1.782 × pT stage (ref. T1–T2) + 1.937 × pN stage (ref. N0) + 1.763 × pM stage (ref. M0) + 2.425 × AJCC stage (ref. I–II) + 1.812 × ISUP grade (ref. 1–2) + 1.714 × *AQP9* expression (ref. Low) for PFS; and another formula: 1.692 × pT stage (ref. T1–T2) + 1.837 × pN stage (ref. N0) + 1.895 × pM stage (ref. M0) + 3.553 × AJCC stage (ref. I–II) + 1.751 × ISUP grade (ref. 1–2) + 1.514 × *AQP9* expression (ref. Low) for OS. The AUC indices for the FUSCC-PFS and FUSCC-OS were 0.823 and 0.828, respectively (*p *< 0.001; Fig. 4a, b).

### Functional annotations and predicted signaling pathways

**Fig. 5:**
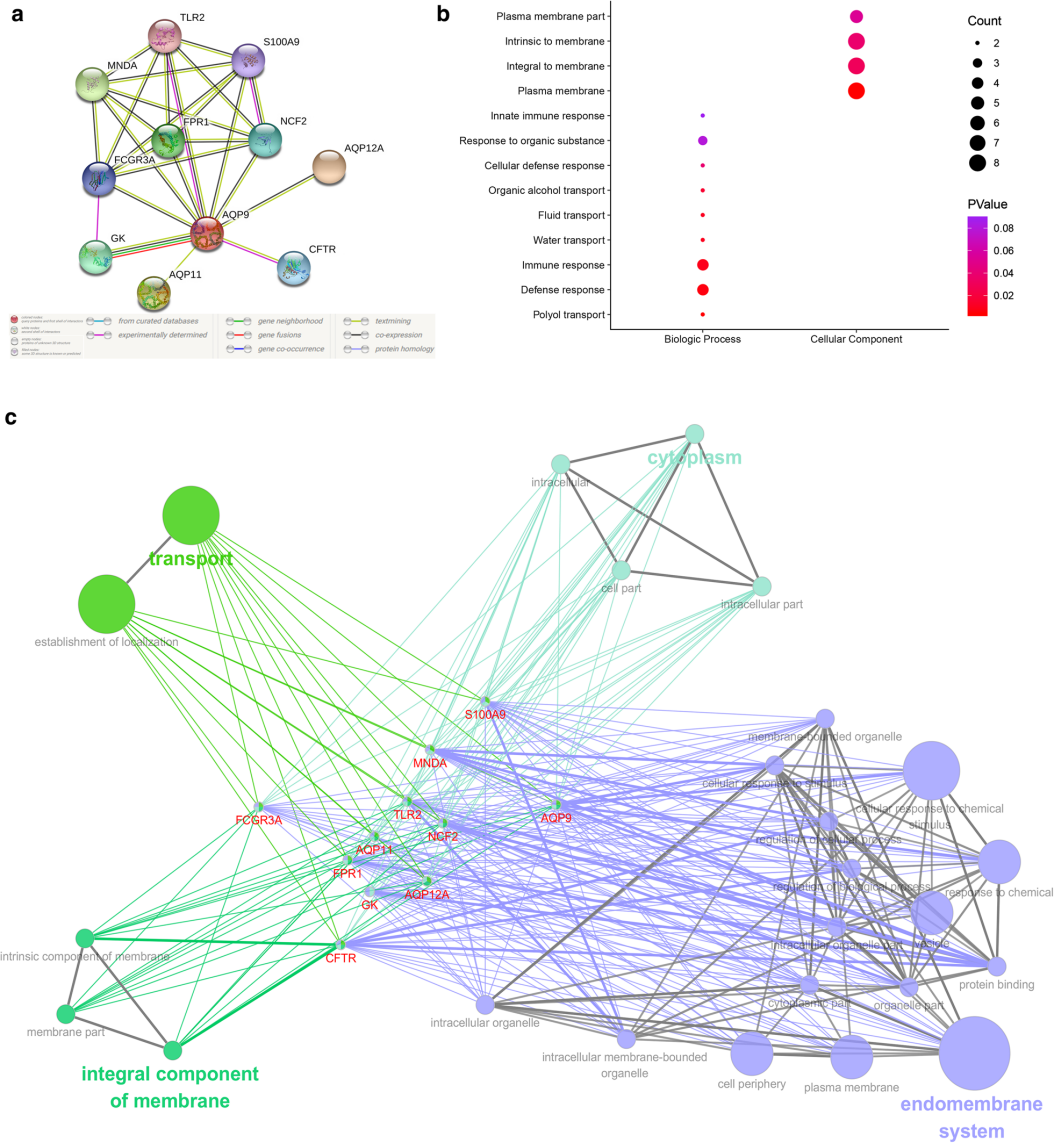
Functional annotations and predicted signaling pathways. **a** The PPI network of *AQP9* was constructed. A network of *AQP9* and its co-expression genes was set up visually. **b** Functional enrichment analyses of a total of 11 involved genes were performed and visualized in bubble chart. Significant genes were significantly involved in polyol transport, defense response, immune response, and markedly participated in plasma membrane, integral to membrane, intrinsic to membrane and plasma membrane part. **c** Functional annotation using ClueGO indicated that changes in the biological processes of the AQP9 were significantly associated with the transport, integral component of membrane and endomembrane system

A network of *AQP9* and its co-expression genes is shown in Fig. 5a. As illustrated in Fig. 5b, functional enrichment analyses of 11 involved genes were performed and the results are visualized in a bubble chart. Significant genes were significantly involved in polyol transport, defense response, and immune response, markedly participated in plasma membrane and were integral to membrane and intrinsic to membrane and the plasma membrane part. As shown in Fig. 5c, functional annotation using ClueGO indicated that changes in the biological processes of the *AQP9* were significantly associated with the transport, integral component of membrane and the endomembrane system. Detailed functional annotations information and the percentage of each term were illustrated in Additional file 2: Figure S2.

### Significant genes and pathways obtained by GSEA

**Fig. 6:**
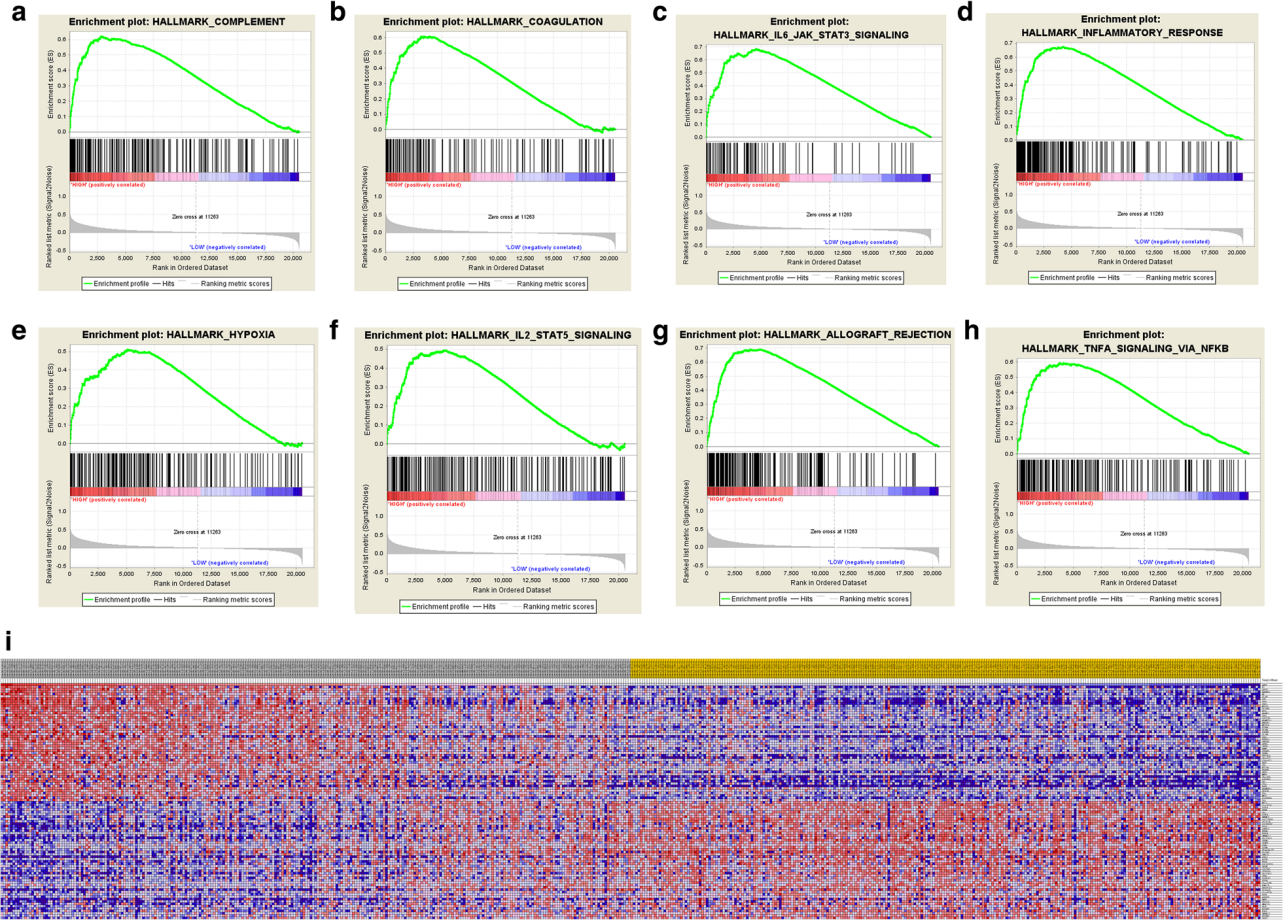
Significant related genes and hallmarks pathways in ccRCC obtained by GSEA. **a**–**h** The most involved significant pathways included complement, coagulation, IL6/JAK/STAT3 signaling, inflammatory response, hypoxia, IL2/STAT5 signaling, allograft rejection and TNF-alpha signaling via NFKB. **i** Transcriptional expression profiles of a total of 100 significant genes with positive and negative correlation were performed in a heat map

A total of 100 significant genes were obtained by GSEA with positive and negative correlations. GSEA was used to perform hallmark analysis for *AQP9*. The results suggested that the most of the involved significant pathways included complement, coagulation, IL6/JAK–STAT3 signaling, inflammatory response, hypoxia, IL2–STAT5 signaling, allograft rejection and TNF-A signaling via NF-κB. The details are shown in Fig. 6a–h. In addition, transcriptional expression profiles of the 100 significant genes are shown by heat map in Fig. 6i.

### Correlation of AQP9 and immune infiltration level

**Fig. 7:**
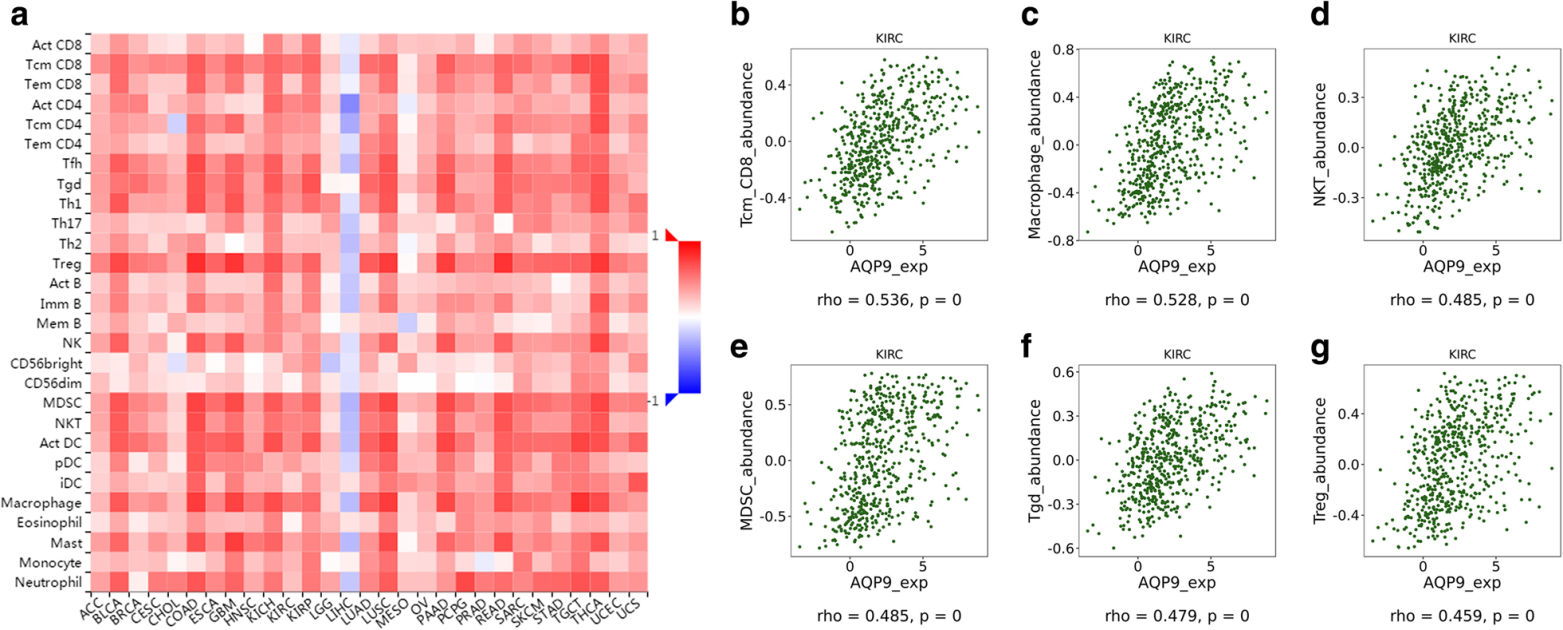
Correlations between expression of AQP9 and TILs across human cancers. **a** Relations between expression of *AQP9* and 28 types of TILs across human heterogeneous cancers. **b**–**g** AQP9 significantly correlated with abundance of central memory CD8 T cells (Tcm_CD8 T cells; rho = 0.536, p < 0.001), macrophage (rho = 0.528, p < 0.001), natural killer T cells (NK T cells; rho = 0.485, p < 0.001), myeloid derived suppressor cells (MDSC; rho = 0.485, p < 0.001), gamma delta T cells (Tgd cells; rho = 0.479, p < 0.001) and Treg (rho = 0.459, p < 0.001)

After determining the prognostic value of *AQP9*, we performed correlation analysis between *AQP9* and immune infiltration level for ccRCC. Elevated *AQP9* was significantly associated with B cell, T cell, monocyte, macrophage, tumor-associated macrophage, and neutrophil cell infiltration (*p *< 0.05), leading to a general increase in immune infiltration. The Spearman’s correlation showed estimated statistical significance between *AQP9* expression and immune cell signature infiltration in Additional file 3: Table S4. Partial correlation and correlation adjusted by tumor purity are also provided. Important signatures of a variety of immune cells including CD8 + T cells, T cells (general), B cells, monocytes, tumor-associated macrophages, M1 macrophages, M2 macrophages, neutrophils, natural killer cells, dendritic cells, Th1, Th2, Tfh, Th17, regulatory T cells (Treg), T cell exhaustion are illustrated in Additional file 3: Table S4. Additionally, we also found significant correlations of AQP9 with 28 types of TILs across human heterogeneous cancers (Fig. 7a). AQP9 significantly correlated with abundance of central memory CD8 T cells (Tcm_CD8 T cells; rho = 0.536, *p *< 0.001), macrophage (rho = 0.528, *p *< 0.001), natural killer T cells (NK T cells; rho = 0.485, *p *< 0.001), myeloid derived suppressor cells (MDSC; rho = 0.485, *p *< 0.001), gamma delta T cells (Tgd cells; rho = 0.479, *p *< 0.001) and Treg (rho = 0.459, *p *< 0.001) in Fig. 7b–g.

## Discussion

Cancer genetics as well as abnormal epigenetic regulation have been found to participate in the progression and tumor environment for ccRCC [6]. The AQP family plays an important role in the development and progression of tumors such as breast cancer [32], nasopharyngeal carcinoma [33], and cervical cancer [34]. Although some members of the AQP family were demonstrated to be carcinogenic in many neoplasms, the prognostic value of *AQP9* in ccRCC had remained to be elucidated. In this study, the expression levels and prognostic values of *AQP9* in ccRCC were evaluated. We observed a significant increase in *AQP9* expression in RCC that was associated with malignant behavior. Our data also indicated that high levels of *AQP9* protein expression correlated with a high risk of recurrence and reduction in patient survival. These results reveal a new way for *AQP9* expression to influence the pathogenesis of RCC through potential DNA damage variants. Functional enrichment and GSEA analysis illustrated that *AQP9* was significantly involved in the most significant hallmarks pathways including inflammatory response, IL6/JAK–STAT3 signal pathway, IL2–STAT5 signal pathway, complement, and TNF-alpha signal pathway in RCC samples.

Inflammation is observed in basic physiological processes and is one of the hallmarks of many neoplasms [35]. Cancer-associated inflammation involves crosstalk between malignant and non-malignant cells in an autocrine and paracrine manner through mediators such as cytokines, chemokines and prostaglandins [36]. In combination with genetic alterations, the inflammatory tumor environment ultimately leads to tumor progression and metastasis [37]. For example, in the absence of the p53 tumor suppressor gene, the inflammatory response that is correlated with epithelial cell senescence significantly promotes transformation and carcinogenesis, which can be inhibited by anti-inflammatory drugs [38]. Treatment with the anti-inflammatory drug dexamethasone also markedly inhibits tumor cell transmission by inhibiting epithelial-to-mesenchymal transition (EMT), a process by which epithelial cells acquire migration and invasion properties [39].

AQP9 in RCC regulates a range of inflammation-related signaling pathways such as IL6/JAK–STAT3, IL2–STAT5 and TNF-alpha signal pathways. Previous research demonstrated that the IL-6/JAK–STAT3 pathway is aberrantly hyperactivated in many carcinomas, and hyperactivation was generally associated with unfavorable clinical prognosis [40]. In the tumor microenvironment, IL-6/JAK–STAT3 signaling promotes proliferation, invasiveness, and metastasis of tumor cells, while strongly suppressing the antitumor immune response [40]. Interleukin-6 (IL-6) is the major cytokine that induces transcriptional acute and chronic inflammation responses and a recurrence prognostic marker for localized ccRCC [41]. In addition, STAT3 is the main mediator of IL-6-induced RCC proliferation [42].

The ability of IL-2 to expand T cells with maintenance of functional activity has been translated into the first reproducible effective human cancer immunotherapies [43]. The use of cytokines from the IL-2 family (also known as the common gamma chain cytokine family) such as IL-2, IL-7, IL-15 and IL-21 to activate the immune system of cancer patients is currently the one of the most important fields of cancer immunotherapy research [43]. Infusion of IL-2 in multiple cycles at distinct doses in patients with metastatic melanoma and RCC has led to the first success in cancer immunotherapy, demonstrating that the immune system can completely eradicate tumor cells under certain conditions [44]. In this study, GSEA analysis indicated that AQP9 regulated the IL2–STAT5 signaling pathway in ccRCC patients. The IL2–STAT5 signaling pathway is involved in immune-related anti-tumor effects, promotes cancer cell proliferation, and interacts with other core cancer-related pathways. Clinical application of IL-2 to exert anti-tumor effects while inhibiting the STAT5 signaling pathway may be an effective treatment strategy for renal cancer.

Decades of research have shown that TNF is a core player in a complex network of cytokines that not only regulates pro-inflammatory responses, but also regulates cellular communication, cell differentiation and cell death including apoptosis and necroptosis [45]. The TNF family receptor Fas was recently found to promote terminal differentiation of CD4^+^ and CD8^+^ T cells, while non-apoptotic Fas signaling induces tumor cell growth and impairs the efficacy of T cell adoptive immunotherapy [46]. The AQP family is also involved in multiple TNA-alpha-induced events [47]. Blocking the non-apoptotic function of these receptors may be a new strategy to enhance anti-tumor immunity.

The relationship between *AQP9* expression and carcinogenesis or prognosis of RCC has been rarely reported. However, it is worth noting that *AQP9* promotes a series of immune responses and tumor environment, which are estimated to be highly expressed in many cancers [6, 48, 49]. Thus, here we used the TCGA database to evaluate the differential *AQP9* expression between tumor and normal tissues, and we validated the prognostic value of *AQP9* in the FUSCC cohort with long follow-up information. Furthermore, to uncover the prognostic significance of *AQP9*, co-regulatory proteins were included in the PPI network. Functional enrichment analysis was measured in hub gene panels. In addition, data from public databases was implemented by GSEA analysis to identify important genes and hallmark pathways, which may shed light on the association that triggers carcinogenesis.

This study has several limitations. First, only transcriptomics expression of *AQP9* with clinical data was analyzed to predict PFS and OS in this study. Although differential AQP9 expression was detected between tumor and normal tissues, the prognostic implication of this finding has not been demonstrated. Second, the underlying mechanisms of signaling pathways in RCC remain unclear, while a serious of function annotations and enrichment analysis were investigated. Future research is required to explore the detailed mechanism between distinct *AQP9* and carcinogenesis of ccRCC and reveal the mechanism of AQP9 in other carcinomas.

## Conclusions

Our study demonstrated that elevated *AQP9* expression was significantly correlated with cancer progression, poor survival and immune infiltrations in ccRCC patients from multiple cohorts. This study provides new and promising insights for subsequent research to elucidate the molecular pathogenesis of ccRCC. Randomized clinical trials and further studies are required to identify the underlying mechanism and clinical applications for ccRCC patients.

## Supplementary information

**Additional file 1: Figure S1.** Differential expression and prognostic value of AQPs (0-11) family number for ccRCC patients from TCGA cohort.

**Additional file 2: Figure S2.** Functional annotations using CluePedia of Cytoscope for AQP9 and its 10 neighbor genes. A. List of the genes count number in different functions in the form of histogramns. B. The proportion of different functional categories, displayed in the form of a pie chart.

**Additional file 3: Table S1.** Clinicopathological characteristics baseline in relation to AQP9 expression status in TCGA cohort. **Table S2.** Univariate Cox logistic regression analysis of PFS in TCGA and FUSCC cohort (PFS: progression-free survival; TCGA: the Cancer Genome Atlas; FUSCC: Fudan university shanghai cancer center). **Table S3.** Univariate Cox logistic regression analysis of OS in TCGA and FUSCC cohort (OS: overall survival; TCGA: the Cancer Genome Atlas; FUSCC: Fudan university shanghai cancer center). **Table S4.** Correlation analysis between AQP9 and immune cell infiltrations in ccRCC samples using TIMER.

## Data Availability

The datasets during and/or analyzed during the current study available from the corresponding author on reasonable request.

## Abbreviations

RCC: renal cell carcinoma
ccRCC: clear cell renal cell carcinoma
FUSCC: Fudan University Shanghai Cancer Center
GO: Gene Ontology
KEGG: Kyoto Encyclopedia of Genes and Genomes
PPI: protein–protein interaction
TCGA: the Cancer Genome Atlas
GEO: gene expression omnibus
PFS: progression-free survival
OS: overall survival
HR: hazard ratio
CI: confidence interval
GSEA: gene set enrichment analysis
