# Supplementary figures and images for: Prognostic implications of Aquaporin 9 expression in clear cell renal cell carcinoma
Source: J Transl Med. 2019 Nov 8;17:363. doi: 10.1186/s12967-019-2113-y (PMC6842264; doi:10.1186/s12967-019-2113-y)

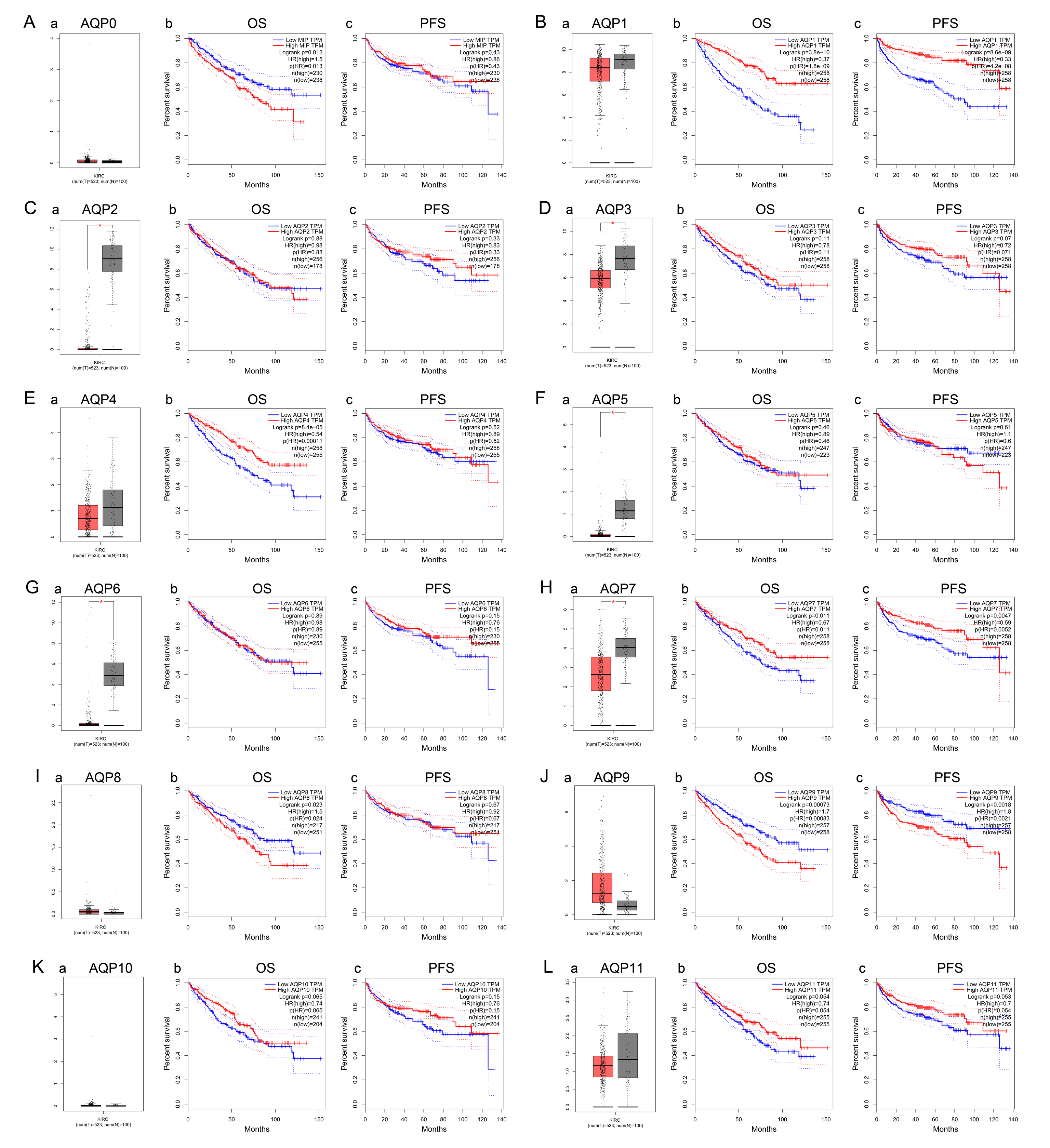

Supplement: Supplementary file 1 — Additional file 1: Figure S1. Differential expression and prognostic value of AQPs (0-11) family number for ccRCC patients from TCGA cohort. [file 12967_2019_2113_MOESM1_ESM.tif]

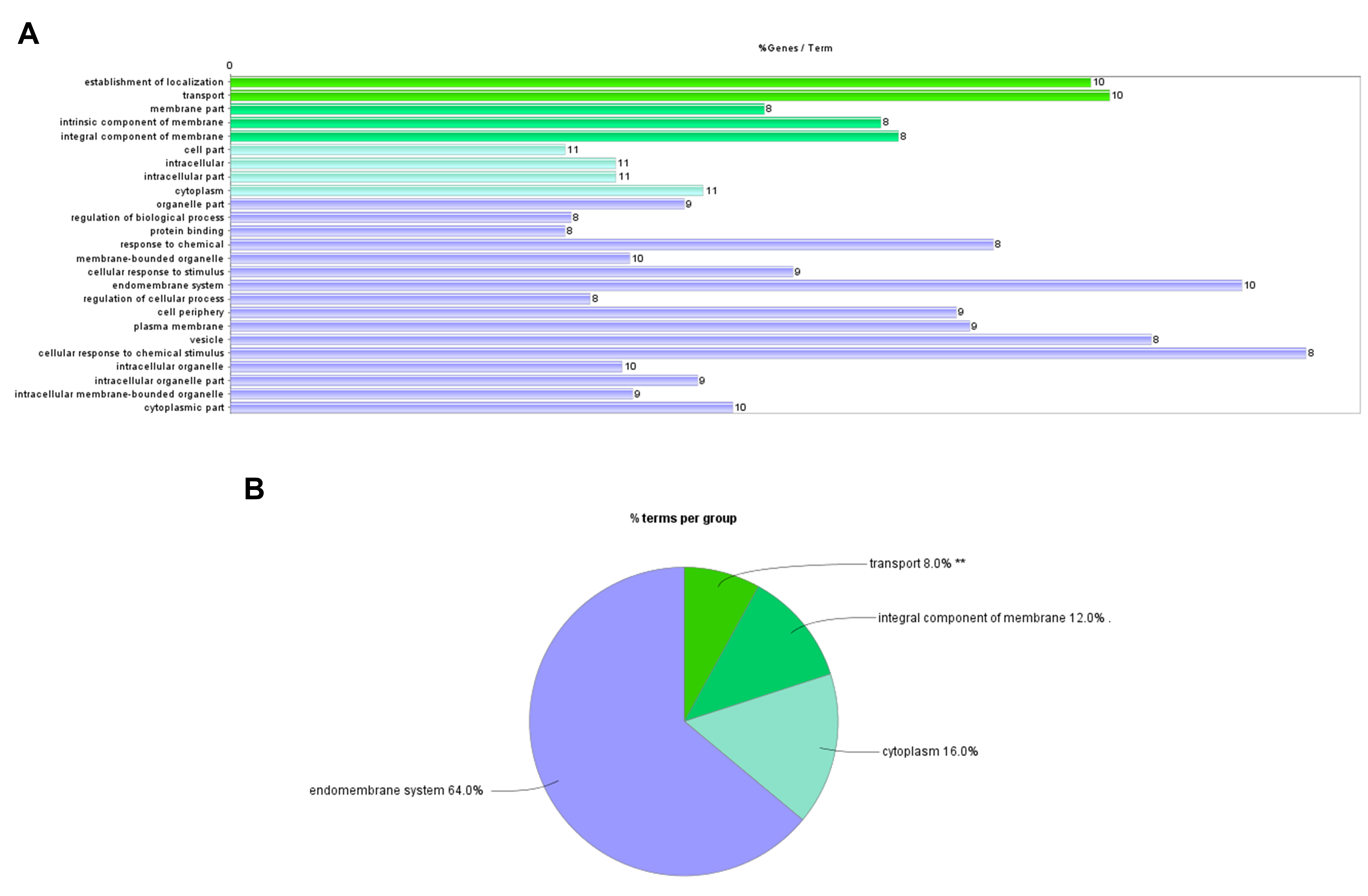

Supplement: Supplementary file 2 — Additional file 2: Figure S2. Functional annotations using CluePedia of Cytoscope for AQP9 and its 10 neighbor genes. A. List of the genes count number in different functions in the form of histogramns. B. The proportion of different functional categories, displayed in the form of a pie chart. [file 12967_2019_2113_MOESM2_ESM.tif]
