# Supplementary material for: Prognostic implications of Aquaporin 9 expression in clear cell renal cell carcinoma
Source: J Transl Med. 2019 Nov 8;17:363. doi: 10.1186/s12967-019-2113-y (PMC6842264; doi:10.1186/s12967-019-2113-y)
Supplement: Supplementary file 3 — Additional file 3: Table S1. Clinicopathological characteristics baseline in relation to AQP9 expression status in TCGA cohort. Table S2. Univariate Cox logistic regression analysis of PFS in TCGA and FUSCC cohort (PFS: progression-free survival; TCGA: the Cancer Genome Atlas; FUSCC: Fudan university shanghai cancer center). Table S3. Univariate Cox logistic regression analysis of OS in TCGA and FUSCC cohort (OS: overall survival; TCGA: the Cancer Genome Atlas; FUSCC: Fudan university shanghai cancer center). Table S4. Correlation analysis between AQP9 and immune cell infiltrations in ccRCC samples using TIMER. [file 12967_2019_2113_MOESM3_ESM.docx]

**Additional file 3: Table S1.** Clinicopathological characteristics baseline in relation to AQP9 expression level in TCGA cohort. (TCGA: The Cancer Genome Atlas)

| Characteristics | TCGA cohort  (N=533) | AQP9 expression | | χ^2^ | *P* |
| --- | --- | --- | --- | --- | --- |
|  |  | High  (N=253) | Low  (N=280) |  |  |
| N (%) |  |  |  |  |  |
| Age |  |  |  | 1.200 | 0.273 |
| ＜60 years | 245 (46.0) | 110 (43.5) | 135 (48.2) |  |  |
| ≥60 years | 288 (54.0) | 143 (56.5) | 145 (51.8) |  |  |
| Gender |  |  |  | 9.793 | **0.002** |
| Male | 345 (64.7) | 181 (71.5) | 164 (58.6) |  |  |
| Female | 188 (35.3) | 72 (28.5) | 116 (41.4) |  |  |
| Laterality |  |  |  | 0.713 | 0.399 |
| Left | 251 (47.1) | 124 (49.0) | 127 (45.4) |  |  |
| Right | 282 (52.9) | 129 (51.0) | 153 (54.6) |  |  |
| pT stage |  |  |  | 38.978 | **<0.001** |
| T1 | 273 (51.2) | 97 (38.3) | 176 (62.9) |  |  |
| T2 | 69 (12.9) | 33 (13.0) | 36 (12.9) |  |  |
| T3 | 180 (33.8) | 114 (45.1) | 66 (23.6) |  |  |
| T4 | 11 (2.1) | 9 (3.6) | 2 (0.7) |  |  |
| pN stage |  |  |  | 11.114 | **0.004** |
| N0 | 240 (45.0) | 115 (45.5) | 125 (44.6) |  |  |
| N1 | 16 (3.0) | 14 (5.5) | 2 (0.7) |  |  |
| Nx | 277 (52.0) | 124 (49.0) | 153 (54.6) |  |  |
| pM stage |  |  |  | 23.159 | **<0.001** |
| M0 | 422 (79.2) | 194 (76.7) | 228 (81.4) |  |  |
| M1 | 79 (14.8) | 53 (20.9) | 26 (9.3) |  |  |
| Mx | 32 (6.0) | 6 (2.4) | 26 (9.3) |  |  |
| AJCC stage † |  |  |  | 39.535 | **<0.001** |
| I | 267 (50.1) | 94 (37.2) | 173 (61.8) |  |  |
| II | 57 (10.7) | 26 (10.3) | 31 (11.1) |  |  |
| III | 123 (23.1) | 74 (29.2) | 49 (17.5) |  |  |
| IV | 86 (16.1) | 59 (23.3) | 27 (9.6) |  |  |
| ISUP grade |  |  |  | 54.691 | **<0.001** |
| G1 | 14 (2.6) | 3 (1.2) | 11 (3.9) |  |  |
| G2 | 229 (43.0) | 77 (30.4) | 152 (54.3) |  |  |
| G3 | 206 (38.6) | 112 (44.3) | 94 (33.6) |  |  |
| G4 | 76 (14.3) | 59 (23.3) | 17 (6.1) |  |  |
| Gx | 8 (1.5) | 2 (0.8) | 6 (2.1) |  |  |

† The AJCC staging system is a classification system developed by the [American Joint Committee on Cancer](https://en.wikipedia.org/wiki/American_Joint_Committee_on_Cancer) for describing the extent of [disease progression](https://en.wikipedia.org/w/index.php?title=Disease_progression&action=edit&redlink=1) in cancer patients. It utilizes in part the [TNM](https://en.wikipedia.org/wiki/TNM_staging_system) scoring system: Tumor size, Lymph Nodes affected, Metastases.

**Additional file 3: Table S2.** Univariate Cox logistic regression analysis of PFS in TCGA and FUSCC cohort (PFS: progression-free survival; TCGA: The Cancer Genome Atlas; FUSCC: Fudan University Shanghai Cancer Center)

| Covariates | TCGA | | |  | FUSCC | | |
| --- | --- | --- | --- | --- | --- | --- | --- |
|  | HR | 95% CI | *P* value |  | HR | 95% CI | *P* value |
| Age | 0.971 | 0.428-2.203 | 0.943 |  | 1.013 | 1.002-1.025 | **0.024** |
| Gender (ref. Male) | 0.243 | 0.072-0.818 | **0.022** |  | 0.762 | 0.563-1.032 | 0.079 |
| BMI (ref. <25kg/m^2^) | - | - | **-** |  | 1.277 | 0.972-1.679 | 0.080 |
| pT stage (ref. T1-T2) | 4.737 | 2.006-11.183 | **<0.001** |  | 7.522 | 5.490-10.307 | **<0.001** |
| pN stage (ref. N0) | 0.048 | 0.000-100 | 0.803 |  | 8.860 | 6.146-12.774 | **<0.001** |
| pM stage (ref. M0) | 8.054 | 3.551-18.266 | **<0.001** |  | 7.842 | 5.730-10.734 | **<0.001** |
| AJCC stage (ref. I-II) | 6.557 | 2.583-16.644 | **<0.001** |  | 9.700 | 7.066-13.316 | **<0.001** |
| ISUP grade (ref. 1-2) | 3.9 | 1.445-10.530 | **0.007** |  | 2.694 | 2.019-3.594 | **<0.001** |
| AQP9 expression (ref. negative) | 8.141 | 2.764-23.976 | **<0.001** |  | 2.593 | 1.948-3.451 | **<0.001** |

**Additional file 3: Table S3.** Univariate Cox logistic regression analysis of OS in TCGA and FUSCC cohort (OS: overall survival; TCGA: The Cancer Genome Atlas; FUSCC: Fudan University Shanghai Cancer Center)

| Covariates | TCGA | | |  | FUSCC | | |
| --- | --- | --- | --- | --- | --- | --- | --- |
|  | HR | 95% CI | *P* value |  | HR | 95% CI | *P* value |
| Age | 1.795 | 1.311-2.456 | **<0.001** |  | 1.015 | 1.002-1.028 | **0.021** |
| Gender (ref. Male) | 1.054 | 0.775-1.434 | 0.737 |  | 0.979 | 0.702-1.364 | 0.898 |
| BMI (ref. <25kg/m^2^) | - | - | - |  | 1.223 | 0.896-1.669 | 0.205 |
| pT stage (ref. T1-T2) | 3.138 | 2.320-4.245 | **<0.001** |  | 9.090 | 6.488-12.737 | **<0.001** |
| pN stage (ref. N0) | 3.380 | 1.795-6.367 | **<0.001** |  | 9.660 | 6.541-14.266 | **<0.001** |
| pM stage (ref. M0) | 3.589 | 2.636-4.886 | **<0.001** |  | 10.118 | 7.171-14.275 | **<0.001** |
| AJCC stage (ref. I-II) | 3.835 | 2.798-5.256 | **<0.001** |  | 13.087 | 9.155-18.708 | **<0.001** |
| ISUP grade (ref. 1-2) | 2.651 | 1.887-3.723 | **<0.001** |  | 3.219 | 2.278-4.547 | **<0.001** |
| AQP9 expression (ref. negative) | 2.262 | 1.657-3.088 | **<0.001** |  | 2.774 | 1.985-3.876 | **<0.001** |

**Additional file 3: Table S4.** Correlation analysis between AQP9 and immune cell infiltrations in ccRCC samples using TIMER.

| Description | Gene markers | AQP9 | | | | |
| --- | --- | --- | --- | --- | --- | --- |
|  |  | None | |  | Purity | |
|  |  | Cor | P |  | Cor | P |
| CD8+ T cell | CD8A | 0.279 | * |  | 0.191 | 0.105 |
|  | CD8B | 0.165 | 0.147 |  | 0.073 | 0.538 |
| T cell (general) | CD3D | 0.323 | ** |  | 0.302 | ** |
|  | CD3E | 0.282 | * |  | 0.22 | 0.0612 |
|  | CD2 | 0.239 | * |  | 0.132 | 0.266 |
| B cell | CD19 | 0.055 | 0.632 |  | 0.002 | 0.989 |
|  | CD79A | 0.384 | *** |  | 0.319 | ** |
| Monocyte | CD86 | 0.314 | ** |  | 0.196 | 0.0958 |
|  | CSF1R | 0.241 | * |  | 0.069 | 0.563 |
| TAM | CCL2 | 0.386 | *** |  | 0.328 | ** |
|  | CD68 | 0.349 | ** |  | 0.248 | * |
|  | IL10 | 0.368 | *** |  | 0.271 | * |
| M1 Macrophage | NOS2 | 0.288 | ** |  | 0.229 | 0.0518 |
|  | IRF5 | 0.241 | * |  | 0.119 | 0.316 |
|  | PTGS2 | 0.363 | ** |  | 0.303 | ** |
| M2 Macrophage | CD163 | 0.393 | *** |  | 0.309 | ** |
|  | VSIG4 | 0.394 | *** |  | 0.292 | * |
|  | MS4A4A | 0.353 | ** |  | 0.254 | * |
| Neutrophils | CEACAM8 | -0.04 | 0.729 |  | -0.057 | 0.632 |
|  | ITGAM | 0.32 | ** |  | 0.184 | 0.119 |
|  | CCR7 | 0.372 | *** |  | 0.296 | * |
| Natural killer cell | KIR2DL1 | 0.102 | 0.373 |  | -0.014 | 0.907 |
|  | KIR2DL3 | 0.149 | 0.191 |  | 0.113 | 0.34 |
|  | KIR2DL4 | 0.174 | 0.124 |  | 0.098 | 0.411 |
|  | KIR3DL1 | 0.042 | 0.712 |  | -0.062 | 0.602 |
|  | KIR3DL2 | 0.143 | 0.209 |  | 0.057 | 0.629 |
|  | KIR3DL3 | 0.072 | 0.526 |  | 0.005 | 0.965 |
|  | KIR2DS4 | 0.073 | 0.524 |  | 0.006 | 0.958 |
| Dendritic cell | HLA-DPB1 | 0.227 | * |  | 0.108 | 0.361 |
|  | HLA-DQB1 | 0.104 | 0.36 |  | 0.022 | 0.852 |
|  | HLA-DRA | 0.219 | 0.0524 |  | 0.09 | 0.448 |
|  | HLA-DPA1 | 0.183 | 0.106 |  | 0.05 | 0.677 |
|  | CD1C | 0.25 | * |  | 0.189 | 0.109 |
|  | NRP1 | 0.349 | ** |  | 0.338 | ** |
|  | ITGAX | 0.339 | ** |  | 0.224 | 0.0563 |
| Th1 | TBX21 | 0.201 | 0.0755 |  | 0.116 | 0.329 |
|  | STAT4 | 0.261 | * |  | 0.188 | 0.111 |
|  | STAT1 | 0.218 | 0.0535 |  | 0.232 | * |
|  | IFNG | 0.291 | ** |  | 0.289 | * |
|  | TNF | 0.247 | * |  | 0.119 | 0.316 |
| Th2 | GATA3 | 0.004 | 0.971 |  | 0.061 | 0.609 |
|  | STAT6 | -0.136 | 0.232 |  | -0.178 | 0.133 |
|  | STAT5A | 0.177 | 0.12 |  | 0.091 | 0.443 |
|  | IL13 | 0.022 | 0.847 |  | 0.134 | 0.258 |
| Tfh | BCL6 | 0.133 | 0.244 |  | 0.125 | 0.292 |
| Th17 | STAT3 | 0.209 | 0.064 |  | 0.162 | 0.17 |
|  | IL17A | 0.181 | 0.11 |  | 0.146 | 0.218 |
| Treg | FOXP3 | 0.199 | 0.079 |  | 0.172 | 0.146 |
|  | CCR8 | 0.293 | ** |  | 0.25 | * |
|  | STAT5B | -0.002 | 0.986 |  | -0.104 | 0.382 |
|  | TGFB1 | 0.225 | * |  | 0.181 | 0.126 |
| T cell exhaustion | PDCD1 | 0.31 | ** |  | 0.224 | 0.0572 |
|  | CTLA4 | 0.238 | * |  | 0.134 | 0.259 |
|  | LAG3 | 0.264 | * |  | 0.199 | 0.0908 |
|  | HAVCR2 | 0.382 | *** |  | 0.296 | * |
|  | GZMB | 0.208 | 0.0656 |  | 0.143 | 0.226 |

TIMER, Tumor IMmune Estimation Resource; TAM, tumor-associated macrophage; Th, T helper cell; Tfh, Follicular helper T cell; Treg, regulatory T cell; Cor, R value of Spearman’s correlation; None, correlation without adjustment. Purity, correlation adjusted by purity

* *P*< 0.05; ** *P*< 0.01; *** *P*< 0.001; **** *P*< 0.0001
